# Family history of premature CHD and risk factor control in patients with a recent ACS

**DOI:** 10.1038/s44325-025-00060-y

**Published:** 2025-07-15

**Authors:** Daniel Yu-Hung Jeng, Haeri Min, Simone Marschner, Desi Quintans, Julie Redfern, Harry Klimis, Graham Hillis, David Brieger, John Atherton, Ravinay Bhindi, Derek Chew, Nicholas Collins, Michael Fitzpatrick, Craig Juergens, Nadarajah Kangaharan, Andrew Maiorana, Michele McGrady, Rohan Poulter, Pratap Shetty, Christian Hamilton-Craig, Peter Thompson, Sandrine Stepien, Amy Von Huben, Shirley Jansen, Clara K. Chow

**Affiliations:** 1 Sydney Medical School, Faculty of Medicine and Health, University of Sydney, Sydney, NSW Australia; 2 Westmead Applied Research Centre, University of Sydney, Westmead, NSW Australia; 3 Faculty of Medicine and Health, University of Sydney, Sydney, NSW Australia; 4 Department of Cardiology, Westmead Hospital, Westmead, NSW Australia; 5 University of Western Australia, Perth, WA Australia; 6 Department of Cardiology, Royal Perth Hospital, Perth, WA Australia; 7 ANZAC Research Institute, Sydney, NSW Australia; 8 Department of Cardiology, Royal Brisbane and Women’s Hospital, Brisbane, QLD Australia; 9 The University of Queensland, Brisbane, QLD Australia; 10 Department of Cardiology, Royal North Shore Hospital, Sydney, NSW Australia; 11 College of Medicine and Public Health, Flinders University, Adelaide, SA Australia; 12 Hunter Medical Research Institute, Newcastle, NSW Australia; 13 Faculty of Medicine, University of New South Wales, Sydney, NSW Australia; 14 Department of Cardiology, Liverpool Hospital, Sydney, NSW Australia; 15 Department of Cardiology, Royal Darwin Hospital, Darwin, NT Australia; 16 Menzies School of Health Research, Darwin, NT Australia; 17 Allied Health Department, Fiona Stanley Hospital, Perth, WA Australia; 18 School of Allied Health, Curtin University, Perth, WA Australia; 19 Department of Cardiology, Royal Prince Alfred Hospital, Sydney, NSW Australia; 20 Department of Cardiology, Sunshine Coast University Hospital, Brisbane, QLD Australia; 21 Department of Cardiology, Wollongong and Shellharbour Hospitals, Wollongong, NSW Australia; 22 Department of Cardiology, Prince Charles Hospital, Brisbane, QLD Australia; 23 Department of Cardiology, Sir Charles Gairdner Hospital, Perth, WA Australia; 24 Curtin Medical School, Curtin University, Perth, WA Australia; 25 The George Institute for Global Health, Sydney, NSW Australia; 26 Department of Vascular and Endovascular Surgery, Sir Charles Gairdner Hospital, Perth, WA Australia; 27 Harry Perkins Institute of Medical Research, Perth, WA Australia

**Keywords:** Acute coronary syndromes, Cardiovascular diseases

## Abstract

Individuals with a family history of premature coronary heart disease (CHD) have increased cardiac morbidity and mortality, which may motivate them to modify their health behaviours. This analysis examines whether patients with self-reported family history of premature CHD experiencing an acute coronary syndrome (ACS) were more likely to have their risk factors and health behaviours under control at 12 months post ACS. Data from the TEXTMEDS study were used to estimate the association between self-reported family history of premature CHD and blood pressure control, LDL cholesterol control, BMI, exercise and smoking status at 12 months post ACS. The study cohort consisted of 1423 participants (mean age 58.0 ± 10.67, 79.2% male), with 556 (39.1%) reporting a family history of premature CHD, while 867 (60.9%) reported no family history. No evidence from this analysis suggests that patients with knowledge of their family history were more likely to achieve better risk factor control. Novel strategies for risk factor control in this high-risk population is required to improve secondary prevention.

## Introduction

Coronary heart disease (CHD) is a condition which confers a significant health burden; it is associated with 17.8 million deaths annually and is also the third leading cause of mortality globally^1^. It is a major cause of mortality among Australians - in 2019 CHD was estimated to underly 11% of all deaths and 42% of all deaths from cardiovascular diseases (CVD)^2^. Individuals with a family history of CHD are at a higher risk of CVD morbidity and mortality compared to those without a family history throughout their lifetime^3^. Furthermore, a family history of premature CHD is associated with an increased risk of disease recurrence such as recurrent myocardial infarction after an acute coronary syndrome (ACS)^4^.

Health-related risk perception refers to an individual’s view on their susceptibility to particular health threats or diseases^5^. It is regarded by many to be an important factor in individuals’ modification of unhealthy behaviours. Evidence suggests that there is a direct correlation between individual risk perceptions and their behaviours in health^6^. Previous studies have shown a direct correlation between perception of cardiovascular risk and health behaviours. Cross-sectional data suggest an association between knowledge and preventative actions including exercise, diet and weight management^7,8^. There is some evidence to indicate that individuals with a family history of premature CHD are aware of their increased risk^9^. Despite this, associations between family history of disease and risk perception were unclear in many other studies, both in general and for CHD specifically^10–12^. This is particularly true for individuals who are disease-free^13^. Hence, establishment of the relationship between family history and perception of their cardiovascular risk is an important factor to consider in the management strategies of this population of patients. This analysis aims to investigate if a self-reported family history of premature CHD in the context of a confirmed ACS diagnosis is associated with behavioural and risk factor control changes. The analysis utilises data from TEXTMEDS (Text Messages to Improve Medication Adherence and Secondary Prevention After Acute Coronary Syndrome) randomised clinical trial^14^.

## Results

This study included 1423 participants from the TEXTMEDS study, having excluded one participant due to non-response to family history of premature CHD question. A total of 556 (39.1%) participants reported a family history of premature CHD. The mean age of participants was lower in those with a family history compared with those without (mean age 56 ± 10.83 years v 59 ± 10.44 years) and women in the sample were more likely to have a positive family history as opposed to not having a family history (61.2% v 46.9%) (Table 1). Baseline BMI and rates of obesity were significantly higher in those with a family history. In addition, there was an increased prevalence of sleep apnoea and depression in the family history group. The proportion of participants prescribed antihypertensive and lipid-lowering agents were similar across the two groups, as was adherence of antihypertensives and statins in the 12-month period the primary study took place (Table 1).

**Table 1:** Cohort characteristics by family history

Characteristics	*N*	Overall, *N* = 1423^a^	FHx, *N* = 556^a^	No FHx, *N* = 867^a^	*p* value^b^
Age	1423	58 (10.67)	56 (10.83)	59 (10.44)	**<0.001**
Gender	1423				**<0.001**
Male		1,127 (79.20%)	407 (73.20%)	720 (83.04%)	
Female		296 (20.80%)	149 (26.80%)	147 (16.96%)	
Ethnicity	1423				**<0.001**
Caucasian		1138 (79.97%)	435 (78.24%)	703 (81.08%)	
Aboriginal/Torres Strait Islander		52 (3.65%)	37 (6.65%)	15 (1.73%)	
Asian		127 (8.92%)	40 (7.19%)	87 (10.03%)	
Arab or Persian		38 (2.67%)	16 (2.88%)	22 (2.54%)	
Other^c^		68 (4.78%)	28 (5.04%)	40 (4.61%)	
BMI	1408	29 (5.48)	30 (5.80)	29 (5.25)	**0.028**
Smoker	1423	378 (26.56%)	160 (28.78%)	218 (25.14%)	0.146
≥10 standard drinks per week	1421	298 (20.97%)	108 (19.46%)	190 (21.94%)	0.292
Employment status	1421				**0.003**
Employed		853 (60.03%)	348 (62.70%)	505 (58.31%)	
Unemployed		178 (12.53%)	81 (14.59%)	97 (11.20%)	
Retired		390 (27.45%)	126 (22.70%)	264 (30.48%)	
Education status	1419				0.868
Year 10 and below		510 (35.94%)	203 (36.58%)	307 (35.53%)	
Year 12 (Higher School Certificate)		242 (17.05%)	96 (17.30%)	146 (16.90%)	
University/Diploma		667 (47.00%)	256 (46.13%)	411 (47.57%)	
Marital Status	1423				0.168
Single		217 (15.25%)	101 (18.17%)	116 (13.38%)	
Married		855 (60.08%)	318 (57.19%)	537 (61.94%)	
Separated		178 (12.51%)	69 (12.41%)	109 (12.57%)	
Widowed		64 (4.50%)	26 (4.68%)	38 (4.38%)	
Defacto		109 (7.66%)	42 (7.55%)	67 (7.73%)	
Income Status	1420				**0.045**
Less than $999 per week/$51,999 per year		447 (31.48%)	193 (34.84%)	254 (29.33%)	
More than $999 per week/$51,999 per year		574 (40.42%)	222 (40.07%)	352 (40.65%)	
No response		399 (28.10%)	139 (25.09%)	260 (30.02%)	
Physical disability	1423	67 (4.71%)	28 (5.04%)	39 (4.50%)	0.735
Prior cardiovascular events	1423	444 (31.20%)	170 (30.58%)	274 (31.60%)	0.727
Myocardial infarction location^d^					
Left Main Coronary	1346	67 (4.98%)	31 (5.87%)	36 (4.40%)	0.226
Left Anterior Descending	1346	855 (63.52%)	337 (63.83%)	518 (63.33%)	0.852
Left Circumflex	1346	536 (39.82%)	212 (40.15%)	324 (39.61%)	0.843
Right Coronary	1346	713 (52.97%)	284 (53.79%)	429 (52.44%)	0.630
Received texts in TEXTMEDS study	1423	715 (50.25%)	291 (52.34%)	424 (48.90%)	0.226
Systolic BP (mmHg)	1420	123 (15.72)	123 (15.31)	123 (15.98)	0.857
Diastolic BP (mmHg)	1420	73 (10.13)	73 (9.80)	73 (10.34)	0.246
LDL-C (mmol/L)	1232	3 (1.09)	3 (1.11)	3 (1.08)	0.271
HDL-C (mmol/L)	1273	1 (0.41)	1 (0.31)	1 (0.46)	0.092
Total cholesterol (mmol/L)	1320	5 (1.36)	5 (1.37)	5 (1.35)	**0.028**
Triglyceride (mmol/L)	1315	2 (1.85)	2 (2.36)	2 (1.40)	**0.004**
Number of antihypertensives prescribed on discharge	1423				0.509
0		87 (6.11%)	36 (6.47%)	51 (5.88%)	
1		374 (26.28%)	137 (24.64%)	237 (27.34%)	
2		962 (67.60%)	383 (68.88%)	579 (66.78%)	
Prescribed statin/lipid-lowering agent on discharge	1423	1,386 (97.40%)	539 (96.94%)	847 (97.69%)	0.485
Hypertension	1423	680 (47.79%)	273 (49.10%)	407 (46.94%)	0.459
Diabetes	1423	340 (23.89%)	145 (26.08%)	195 (22.49%)	0.138
Obesity	1408	544 (38.64%)	246 (44.65%)	298 (34.77%)	**<0.001**
Hyperlipidaemia	1423	709 (49.82%)	293 (52.70%)	416 (47.98%)	0.093
Sleep apnoea	1423	154 (10.82%)	78 (14.03%)	76 (8.77%)	**0.002**
History of depression	1423	265 (18.62%)	125 (22.48%)	140 (16.15%)	**0.003**
Chronic kidney disease	1422	37 (2.60%)	12 (2.16%)	25 (2.88%)	0.507
SF12 quality of life physical health score	1423	25 (6.36)	25 (6.99)	26 (5.88)	**0.001**
SF12 quality of life mental health score	1423	13 (3.17)	13 (3.53)	13 (2.91)	**0.007**
Anxiety	828	116 (14.01%)	53 (16.31%)	63 (12.52%)	0.153
Exercising regularly	1415	902 (63.75%)	353 (63.83%)	549 (63.69%)	>0.999
Adherence to beta blockers at 6/12 months^e^	1193	1001 (83.91%)	390 (83.33%)	611 (84.28%)	0.725
Adherence to ACEi/ARB at 6/12 months^e^	1256	986 (78.50%)	387 (79.47%)	599 (7.89%)	0.555
Adherence to statins at 6/12 months^e^	1322	1255 (94.93%)	478 (93.73%)	777 (95.69%)	0.145

Bold values indicates statistical significance at 5% (*p* ≤ 0.05).

*FHx* family history, *BMI* body mass index, *BP* blood pressure, *LDL-C* low-density lipoprotein cholesterol, *HDL-C* high-density lipoprotein cholesterol, *SF12* 12-item Short Form Survey, *ACEi* Angiotensin-converting enzyme inhibitor, *ARB* Angiotensin receptor blocker, *SD* standard deviation.

^a^Mean (SD); *n* (%).

^b^Welch Two Sample t-test; Pearson’s Chi-squared test.

^c^Includes Black African and Coloured African (Sub-Saharan Africa).

^d^Infarction location defined by stenosis greater than 50% in the specified vessel.

^e^Adherence is defined as > 80% adherent to the particular medication at both the 6- and 12-month follow-up.

### Outcomes by family history

**Fig. 1:**
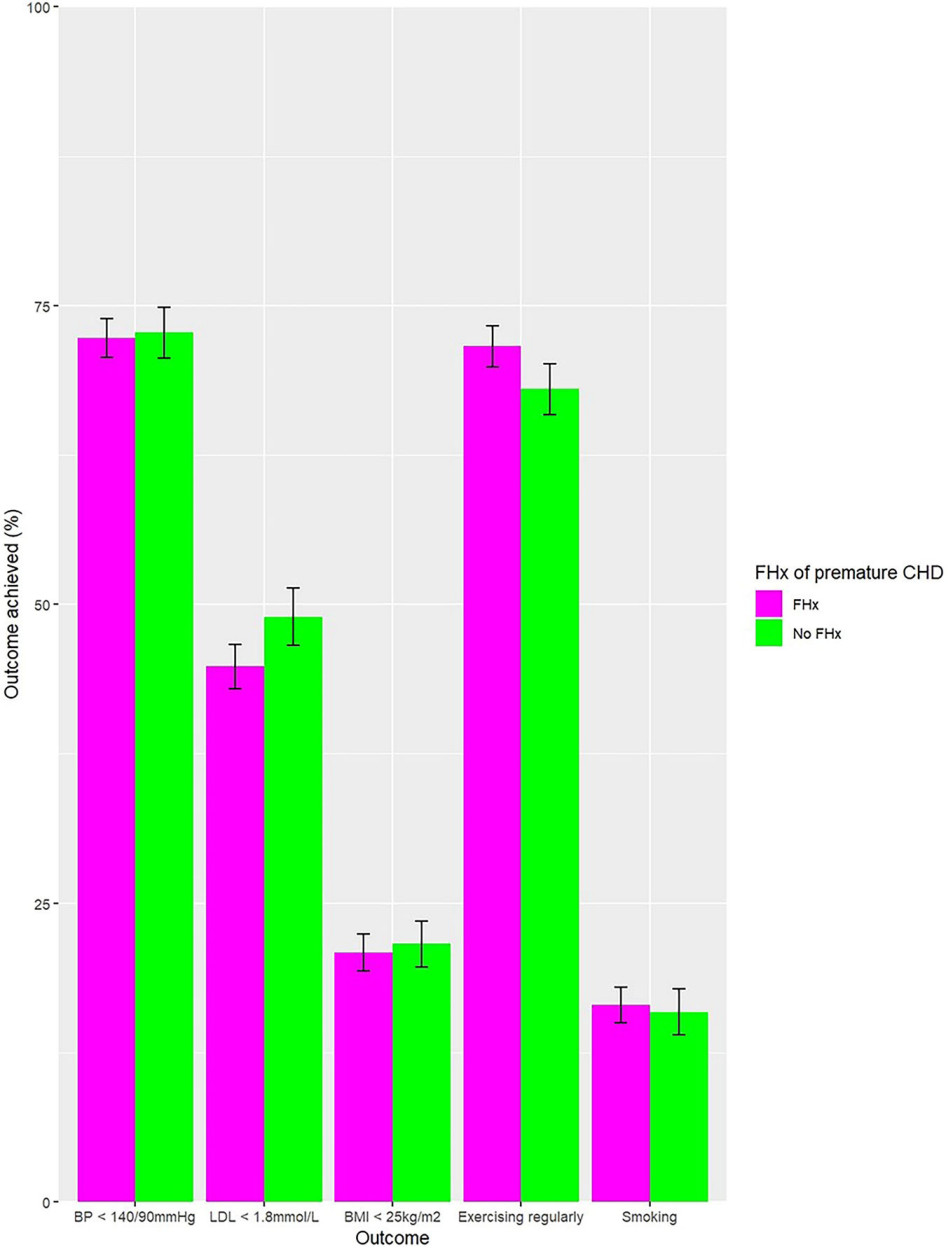
Multiple bar chart of percentage of outcome achievement for each outcome of interest at 12 months follow-up by family history of premature CHD. Errors bars represent standard error. Abbreviations used: FHx family history, CHD coronary heart disease, BP blood pressure, LDL low-density lipoprotein, BMI body mass index.

A multiple bar chart of the percentage of outcome achievement by family history has been presented (Fig. 1). Initial unadjusted logistic regression models suggested no association between family history status and any of the five outcomes at 12 months (Table 2).

**Table 2:** Simple and multiple logistic regression models for relationship of family history of premature CHD and target risk factor outcome variables achieved at 12 months post-acute coronary syndrome

Outcome	*N*	Unadjusted model OR (95% CI)	*p* value	Adjusted model^a^ OR (95% CI)	*p* value
BP < 140/90 mmHg	1214	0.98 (0.76, 1.27)	0.874	0.92 (0.68, 1.24)	0.581
LDL < 1.8 mmol/L	1156	0.85 (0.67, 1.08)	0.173	0.92 (0.70, 1.21)	0.555
BMI < 25 kg/m^2^	1164	0.96 (0.71, 1.28)	0.766	0.99 (0.59, 1.64)	0.954
Exercising regularly^b^	1205	1.18 (0.92, 1.53)	0.191	1.29 (0.97, 1.73)	0.086
Smoking^@^	1248	1.04 (0.76, 1.42)	0.792	0.88 (0.59, 1.31)	0.546

Each multiple logistic regression model has been adjusted by age, sex, intervention in the primary study, baseline value of the outcome and the specific selected variables in each outcome of interest.

*OR* odds ratio, *CI* confidence interval, BP blood pressure, *LDL* low-density lipoprotein, *BMI* body mass index.

^a^Adjusted for age, sex, treatment in primary study, baseline value of the outcome of interest and specific selected variables for each outcome.

^b^GPAQ score equal or greater than 600 and smoking status at 12 months post-discharge @ Current smoker; non-smokers and former smokers are both defined as negative for smoking.

In the final model for achieving target blood pressure, history of hypertension was the only covariate independently associated with the outcome. In the adjusted model, no association was found between family history and achieving blood pressure target at 12 months (adjusted odds ratio (aOR) = 0.92, 95% CI = 0.68, 1.24). Smoking, drinking, marital status, physical disability, baseline HDL cholesterol, lipid agent use, diabetes and hyperlipidaemia were associated with achieving LDL cholesterol target. In the adjusted model no association was found between family history and achieving LDL cholesterol target (aOR = 0.92, 95% CI = 0.70, 1.21). In the final model for achieving target BMI, ethnicity, employment, baseline HDL cholesterol, baseline triglyceride, hypertension, diabetes, hyperlipidaemia, sleep apnoea and obesity were independent predictors. In the adjusted model there was no association between family history and achieving BMI < 25 (aOR = 0.99, 95% CI = 0.59, 1.64). The final model for achieving exercise target, included BMI, alcohol consumption, employment, education, marital status, prior cardiovascular events, hypertension, obesity, depression, chronic kidney disease and SF12 quality of life physical health scores. In the final adjusted model, no association was found between family history and exercise target (aOR = 1.29, 95% CI = 0.97, 1.73). The final model for achieving non-smoking target included education level, marital status and depression and no association was found for family history and non-smoking at 12 months (aOR = 0.88, 95% CI = 0.59, 1.31) (Table 2). Similar findings were found on all four outcomes excluding smoking when analysed with a continuous regression model. Multiple comparison adjustment was not performed due to the exploratory nature of this analysis that required further hypothesis testing had there been clinically significant outcomes.

## Discussion

In this secondary analysis of the TEXTMEDS study, we found no association between family history of CHD and risk factor control (blood pressure, LDL cholesterol, BMI, exercise, smoking) at 12 months after their ACS. We found that roughly a third of the cohort who have had an ACS had a family history of premature CHD. Participants with a family history of premature CHD were younger and more likely to be female but were otherwise like those without a family history of CHD, with comparable levels of hypertension, hyperlipidaemia, exercise, BMI and smoking.

Our findings suggests that knowledge of family history may not influence behaviour change with respect to cardiovascular risk factors in patients with a recent ACS, despite the expectation that patients with knowledge of their family history perhaps should perceive their risk to be higher. The absence of an improvement in risk factor control compared to the participant group without a family history after baseline adjustment is consistent with some prior studies studying the association of only family history or only prior ACS to behavioural changes. In study populations with a family history of premature CVD, cross-sectional and case-control data examined by Ton et al. found that approximately half of the siblings and offspring of patients that suffered from CHD believed their risk of CHD to be equal to or less than the general population^11^. Ter Hoeve demonstrated that in patients with a past ACS controllable risk factors e.g. cholesterol, BMI were often underestimated, with external factors being an emphasis e.g. stress, work as the cause of their ACS; even in those who identified controllable risk factors as a major contributor, rates of programme participation to control these factors remained suboptimal^15^. Andersson et al. explored the effects that a personal experience of disease or a family history of CVD may have on a variety of risk factors i.e. smoking, exercise, BMI levels^16^. While a difference was seen in smoking, as a result of a personal experience to illness, there were no differences observed due to family history. Elis et al. investigated cross-sectional data consisting of mostly young adults, and results showed that smoking and exercise habits remain unchanged between groups with and without a family history of CVD^17^.

Risk perception from a family history, similarly, did not associate itself with risk factor and behaviour management. Imes and Lewis’ systematic review consisting of 25 studies between 1986 and 2012 of mostly cross-sectional studies concluded that both an awareness of family history and the perception of one’s own risk for CVD was not sufficient to drive health behaviour changes to a significant level^10^. This was true for both smoking and exercise, where Hunt’s study found that apart from the youngest cohort of participants interviewed (around age 23), those with a family history of CVD, while aware of their increased risk, did not think that reducing smoking and increasing exercise was of more importance to them^18^. Also mentioned in the systematic review was Kavanagh’s study; attempts at adhering to exercise and weight loss regimes were suboptimal at 50% or lower in participants who had a first degree relative with premature CHD^19^. Tavares, Oliveira and Lopes’ study consisting of questionnaires delivered to individuals within the Portuguese general population further failed to show any dissimilarity in care-seeking and health behaviour between individuals with and without a family history^20^. Studies investigating differences in the same biological factors evaluated in this study according to the presence of family history reported no significant differences in the achievement of systolic blood pressure, diastolic blood pressure and LDL cholesterol targets^21,22^.

Conversely, studies involving an intervention targeting at improving risk factor control specifically in individuals with family history demonstrated efficacy. Goldfarb et al. conducted a systematic review and meta-analysis on primary screening and intervention of cardiac disease in individuals with a family history of cardiac disease, which suggested that active intervention in these individuals may be effective for risk factor or health behaviour modification^23^. Similarly, a text message randomised controlled trial undertaken by Ruffin et al. targeting individuals’ familial risk of six common diseases including CHD showed modest changes in lifestyle factors with increases in exercise and diet habits^24^. However, these positive studies consisted of a targeted effort to increase risk perception in the specific population with a family history, rather than just evaluating the sole effect of family history on health behaviour and risk factor control over time. On the other hand, this can also mean that familial risk is possibly a contributing factor to an individual’s amenability to modify their own lifestyle choices.

The underlying reason behind this lack of association between family history and risk factor control is likely to be multifactorial and complex. One of the possible explanations may be that the interpretation of what a family history of CHD entails can differ between the general public and that of clinicians. Family members of premature CHD patients may attempt to find dissimilarities between their lifestyles and attribute premature CHD to these external factors, rather than an intrinsic tendency to develop CHD^25^. It is also possible that the degree of risk perceived from having prior adverse cardiovascular events is similar or greater than that from a family history, which can result in no appreciable differences in risk factor control between the two groups of participants^26^. It is also possible that inherently, risk factor control is more difficult to achieve in these individuals in which poor control of these risk factors is what contributed originally to a family history of premature CHD. It is worth noting that an inability, rather than an unwillingness to control risk factors may be relevant in participants with a family history; factors such as socioeconomic disadvantage is associated with poorer risk factor management^27,28^, and this may contribute to a higher frequency of premature CHD in family members.

The main strength in the current study is that the data was obtained from a randomised clinical trial with uniformly collected data points on risk factor outcomes for all participants at 12 months. These data were collected from multiple states within Australia, making the results more generalisable within the country. A limitation of this study is that family history is obtained by self-report and hence may have been over or under-reported, however we are interested in patients who know their family history so self-reporting is appropriate in this case. There were missing data at the 12-month follow-up timepoint which resulted in exclusion of individuals in the final data analysis. This was 14.7% for blood pressure, 18.8% for LDL cholesterol, 15.4% for exercise and 18.3% for BMI (Supplementary Tables 2–6). Comparison of individuals with and without missing values suggested that the former group of individuals were less likely to be employed or receive higher education. They were also more associated with comorbidities at baseline such as physical disability, diabetes, sleep apnoea and depression. This may introduce selection bias into the final model which could have affected the results. A further sensitivity analysis was conducted where each missing value for each outcome were imputed and replaced with a negative outcome which showed no changes in the results. In-depth interviews would be useful for future studies to fully understand whether family history acts as a catalyst to improve cardiovascular risk factors through risk factor control and lifestyle behaviours.

This study found evidence that among patients who have experienced an ACS, a family history of premature CHD is not associated with differences in the control of cardiovascular risk factors a year post ACS. Given the high lifetime risk that a history of premature CHD is associated with, further research is needed to understand how to improve the cardiovascular risk factor management and control of this group. Monitoring and managing risk factors are important in all ACS patients and particularly important in those with a family history of premature CHD due to their elevated risk.

## Methods

The TEXTMEDS study recruited 1424 participants as described within the primary study protocol paper^29^. Briefly, the primary study was a single-blind randomised controlled trial of patients after an episode of ACS. Patients either received usual secondary prevention care according to the treating clinician (control) or received additional weekly text messages with medication and lifestyle advice for the time period of a year. The primary study discovered that medical adherence did not improve as a result of the additional intervention but had small effects on BMI and dietary habits.

As per the primary study, patients were screened from metropolitan and rural hospitals and medical centres across five states in Australia with a confirmed diagnosis of an ACS on admission to the hospital. This was defined as follows:

1. Acute myocardial infarction defined by the Third Universal Definition of myocardial infarction^30^ OR
2. Admitted to hospital with symptoms consistent with cardiovascular ischaemia but not meeting requirements of the criteria above and one of (a) angiography suggestive of CHD (narrowing of at least one coronary artery greater than 50%) (b) prior coronary artery bypass graft surgery (c) prior percutaneous coronary intervention.

Additional inclusion criteria were based on participants’ ability to give consent, possession of a mobile device capable of text function, adequate command of English, as well as a life expectancy of more than 6 months. This analysis includes participants from TEXTMEDS who provided data regarding a family history of premature CHD. Consent has been obtained from the participants at the time of the primary study for the data collected to be discussed and/or published in peer-reviewed journals.

### Statistical power

We consider an improvement of 10% to be clinically significant. To detect a relative risk ratio of 10% meeting the blood pressure target from a baseline of 70% in those without a family history to 77% of those with a family history, the main study of 1423 participants will have 85% power.

### Data collection

Data collection has been previously described^29^. In summary, data were collected at baseline and follow-up assessments were at 6 months and 12 months. The baseline and 12-month visits were conducted in-person, while the 6-month visit was conducted via telephone. Baseline family history of premature CHD was defined as a diagnosis of CHD in at least one first degree male relative under the age of 55, or one first degree female relative under the age of 65 years^31^.

In this study the outcomes are achieving target blood pressure, LDL cholesterol, body mass index (BMI) and smoking status at 12 months. The data for these five outcomes were not collected at the 6-month follow-up appointments. Thresholds defining risk factor control were blood pressure lower than 140/90 mmHg, LDL cholesterol concentration lower than 1.8 mmol/L and BMI lower than 25 kg/m^2^; these are based on the standard definition of hypertension, optimal LDL cholesterol levels for patients with past cardiovascular events and the definition of overweight as follows:

Hypertension: grade 1—140–159 mmHg systolic or 90–99 mmHg diastolic, grade 2— 160–179 mmHg systolic or 100–109 mmHg diastolic, grade 3—180 mmHg systolic or 110 mmHg diastolic and above^32^.

LDL cholesterol: For patients with past ACS, the recommended LDL-C goal is ≤1.8 mmol/L (≤70 mg/dL) while commenced on anti-lipid/statin therapy^33,34^.

BMI: Based on current WHO definition of overweight (BMI ≥ 25)^35^.

GPAQ (Global Physical Activity Questionnaire) score ≥ 600 MET-minutes per week was used to identify participants who were exercising regularly. A GPAQ score of 600 is equal to 75 min of vigorous activity, 150 min of moderate activity or an equivalent combination of both reaching 600 MET-minutes per week^36^.

### Statistical method

Data analysis has been performed on R version 4.3.1. Family history and each outcome of interest was compared initially with unadjusted Welch Two Sample *t* tests and Pearson’s Chi-squared tests. Statistically relevant baseline factors within the TEXTMEDS study dataset were identified for each outcome of interest using univariate logistic regression (Supplementary Tables 2–6). The statistical method of LASSO (Least Absolute Shrinkage and Selection Operator) was then utilised to further identify and narrow down relevant covariates^37^. To summarise briefly, this statistical method selects only for covariates that can better predict each of the outcomes being investigated in our study. The covariates that remain within the model after the LASSO method were then included and adjusted for within the final model for each outcome. The final model for each outcome was adjusted by age, sex, intervention in the primary study, baseline value for each outcome and the covariates that were identified using LASSO. In addition, analyses were conducted using the continuous outcome variables in a regression model for all outcomes except smoking status.

This project was approved by the Western Sydney Local Health District Human Research Ethics Committee (approval HREC2012/12/4.1 [3648] AU RED HREC/13/WMEAD/15); the primary study has been listed on the Australian New Zealand Clinical Trials Registry (ACTRN12613000793718).

## Supplementary information

Supplementary Materials

## Data Availability

Data are available on request and approval by the TEXTMEDS investigators.

## Code availability

Code for the data analysis is available on request and approval by the corresponding authors.
