# Supplementary material for: Family history of premature CHD and risk factor control in patients with a recent ACS
Source: NPJ Cardiovasc Health. 2025 Jul 15;2:37. doi: 10.1038/s44325-025-00060-y (PMC12912435; doi:10.1038/s44325-025-00060-y)
Supplement: Supplementary file 1 — Supplementary Materials [file 44325_2025_60_MOESM1_ESM.pdf]

Supplementary Materials:

Supplementary Table 1. Rates of outcomes at baseline visit and at 12-month follow-up in participants with and without a family history of premature coronary heart disease.

	Baseline visit			12-month follow-up		
**Outcome**	**N**	**FHx, N = 556**[1]	**No FHx, N = 867**[1]	**N**	**FHx, N = 556**[1]	**No FHx, N = 867**[1]
**BP < 140/90mmHg**	1,420	473 (85.23%)	721 (83.35%)	1,214	329 (72.31%)	552 (72.73%)
**LDL < 1.8mmol/L**	1,232	86 (17.95%)	153 (20.32%)	1,156	195 (44.83%)	353 (48.96%)
**BMI < 25kg/m^2**	1,408	127 (23.05%)	153 (20.32%)	1,164	93 (20.85%)	155 (21.59%)
**Exercising regularly***	1,415	353 (63.83%)	549 (63.69%)	1,205	330 (71.58%)	506 (68.01%)
**Smoking**[@]	1,423	160 (28.78%)	218 (25.14%)	1,248	78 (16.46%)	123 (15.89%)

[1] **n (%)**

* GPAQ score equal or greater than 600 and smoking status at 12 months post-discharge @ Current smoker; non-smokers and former smokers are both defined as negative for smoking.

Abbreviations used: FHx = family history, BP = blood pressure, LDL = low-density lipoprotein, BMI = body mass index

Supplementary Table 2. Univariate logistic regression for possible association of covariates with blood pressure outcome.

Covariates	N	OR[1]	95% CI[1]	p-value
**Ethnicity (Caucasian)**	1,214	1.00	0.73, 1.36	0.997
**BMI**	1,205	0.96	0.94, 0.98	<0.001
**Smoker**	1,214	1.54	1.14, 2.11	0.005
**>=10 standard drinks per week**	1,213	0.82	0.61, 1.11	0.202
**Employed full-time/part-time**	1,212	1.34	1.04, 1.74	0.024
**Education Level**	1,211			0.208
**Year 10 or under**		—	—	
**Year 12**		1.34	0.92, 1.97	
**University/Diploma or above**		1.23	0.93, 1.63	
**Married/Defacto**	1,214	0.93	0.70, 1.22	0.582
**Income greater than 1,000 AUD/week**	1,213	1.19	0.91, 1.56	0.202
**Physical disability**	1,214	0.97	0.53, 1.88	0.927
**Prior cardiovascular events**	1,214	0.76	0.58, 1.00	0.046
**LDL < 1.8 mmol/L**	1,058	0.67	0.49, 0.94	0.020
**HDL > 1.0 mmol/L**	1,092	0.95	0.73, 1.24	0.691
**Triglyceride (mmol/L)**	1,129	0.93	0.86, 1.00	0.064
**Number of antihypertensives prescribed on discharge**	1,214			0.022
**0**		—	—	
**1**		0.41	0.19, 0.80	
**2**		0.42	0.20, 0.79	
**Hypertension**	1,214	0.35	0.27, 0.46	<0.001
**Diabetes**	1,214	0.81	0.60, 1.09	0.157
**Obesity**	1,205	0.72	0.55, 0.93	0.012
**Hyperlipidaemia**	1,214	0.67	0.52, 0.86	0.002
**Sleep apnoea**	1,214	0.91	0.61, 1.39	0.655
**History of depression**	1,214	1.19	0.85, 1.69	0.314

**Chronic kidney disease**	1,213	0.34	0.17, 0.71	0.004
**SF12 quality of life mental health score**	1,214	1.01	0.97, 1.05	0.773
**Exercising regularly**	1,207	1.09	0.84, 1.42	0.505

[1] OR = Odds Ratio, CI = Confidence Interval

Note: blood pressure criteria defined as lower than 140/90mmHg in both systolic and diastolic pressure at 12 months post-discharge.

Abbreviations used: OR = odds ratio, CI = confidence interval, BMI = body mass index, LDL = low-density lipoprotein, HDL = high-density lipoprotein, SF12 = 12-item Short Form Survey

Supplementary Table 3. Univariate logistic regression for possible association of covariates with LDL-C outcome.

Covariates	N	OR[1]	95% CI[1]	p-value
**Ethnicity (Caucasian)**	1,156	0.81	0.61, 1.07	0.138
**BMI**	1,145	1.01	0.99, 1.03	0.423
**Smoker**	1,156	0.57	0.43, 0.75	<0.001
**>=10 standard drinks per week**	1,155	0.58	0.43, 0.78	<0.001
**Employed full-time/part-time**	1,155	0.79	0.62, 1.00	0.047
**Education Level**	1,153			0.754
**Year 10 or under**		—	—	
**Year 12**		1.14	0.81, 1.59	
**University/Diploma or above**		1.06	0.82, 1.38	
**Married/Defacto**	1,156	1.28	0.99, 1.64	0.057
**Income greater than 1,000 AUD/week**	1,155	1.09	0.85, 1.41	0.502
**Physical disability**	1,156	1.70	0.93, 3.17	0.084
**Prior cardiovascular events**	1,156	1.04	0.81, 1.33	0.770
**BP < 140/90**	1,154	0.98	0.72, 1.33	0.881
**HDL > 1.0 mmol/L**	1,050	0.80	0.63, 1.02	0.078
**Triglyceride (mmol/L)**	1,078	0.93	0.85, 1.02	0.122
**Prescribed statin/lipid-lowering agent on discharge**	1,156	2.60	1.07, 7.25	0.034
**Hypertension**	1,156	1.26	1.00, 1.59	0.048
**Diabetes**	1,156	1.89	1.43, 2.51	<0.001
**Obesity**	1,145	1.02	0.80, 1.30	0.863
**Hyperlipidaemia**	1,156	0.78	0.62, 0.98	0.037
**Sleep apnoea**	1,156	1.10	0.75, 1.62	0.621
**History of depression**	1,156	0.77	0.57, 1.05	0.103
**SF12 quality of life mental health score**	1,156	0.99	0.95, 1.03	0.548
**Exercising regularly**	1,149	0.95	0.74, 1.20	0.644

[1] **OR = Odds Ratio, CI = Confidence Interval**

Note: LDL cholesterol criteria defined as lower than 1.8mmol/L at 12 months post-discharge.

Abbreviations used: OR = odds ratio, CI = confidence interval, BMI = body mass index, BP = blood pressure, LDL = low-density lipoprotein, HDL = high-density lipoprotein, SF12 = 12-item Short Form Survey

Supplementary Table 4. Univariate logistic regression for possible association of covariates with BMI outcome.

Covariates	N	OR[1]	95% CI[1]	p-value
**Ethnicity (Caucasian)**	1,164	0.64	0.46, 0.89	0.009
**Smoker**	1,164	1.16	0.84, 1.59	0.353
**>=10 standard drinks per week**	1,163	0.86	0.60, 1.21	0.392
**Employed full-time/part-time**	1,162	0.61	0.46, 0.82	<0.001
**Education Level**	1,162			0.225
**Year 10 or under**		—	—	
**Year 12**		1.41	0.93, 2.11	
**University/Diploma or above**		1.23	0.89, 1.70	
**Married/Defacto**	1,164	0.89	0.66, 1.20	0.433
**Income greater than 1,000 AUD/week**	1,163	0.78	0.58, 1.04	0.094
**Physical disability**	1,164	0.73	0.31, 1.50	0.410
**Prior cardiovascular events**	1,164	0.98	0.72, 1.32	0.887
**BP < 140/90**	1,164	1.48	0.99, 2.29	0.058
**LDL < 1.8 mmol/L**	1,009	0.75	0.50, 1.11	0.155
**HDL > 1.0 mmol/L**	1,042	1.84	1.37, 2.48	<0.001
**Triglyceride (mmol/L)**	1,079	0.54	0.44, 0.65	<0.001
**Hypertension**	1,164	0.52	0.39, 0.69	<0.001
**Diabetes**	1,164	0.45	0.30, 0.66	<0.001
**Obesity**	1,164	0.01	0.00, 0.03	<0.001
**Hyperlipidaemia**	1,164	0.72	0.54, 0.95	0.020
**Sleep apnoea**	1,164	0.17	0.07, 0.37	<0.001
**History of depression**	1,164	0.84	0.57, 1.21	0.354
**Chronic kidney disease**	1,163	1.42	0.58, 3.13	0.419
**SF12 quality of life mental health score**	1,164	0.98	0.94, 1.03	0.414
**Exercising regularly**	1,157	1.02	0.76, 1.37	0.889

[1] OR = Odds Ratio, CI = Confidence Interval

Note: BMI criteria defined as lower than 25 at 12 months post-discharge.

Abbreviations used: OR = odds ratio, CI = confidence interval, BMI = body mass index, BP = blood pressure, LDL = low-density lipoprotein, HDL = high-density lipoprotein, SF12 = 12-item Short Form Survey

Supplementary Table 5. Univariate logistic regression for possible association of covariates with exercise outcome.

Covariates	N	OR[1]	95% CI[1]	p-value
**Ethnicity (Caucasian)**	1,205	0.93	0.68, 1.26	0.650
**BMI**	1,197	0.96	0.94, 0.98	<0.001
**Smoker**	1,205	1.11	0.83, 1.48	0.480
**>=10 standard drinks per week**	1,204	1.72	1.24, 2.42	<0.001
**Employed full-time/part-time**	1,204	2.08	1.62, 2.67	<0.001
**Education Level**	1,202			<0.001
**Year 10 or under**		—	—	
**Year 12**		1.35	0.95, 1.92	
**University/Diploma or above**		1.82	1.38, 2.39	
**Married/Defacto**	1,205	1.45	1.12, 1.88	0.005
**Income greater than 1,000 AUD/week**	1,204	1.33	1.03, 1.73	0.032
**Prior cardiovascular events**	1,205	0.67	0.52, 0.87	0.003
**BP < 140/90**	1,203	0.93	0.66, 1.31	0.695
**Hypertension**	1,205	0.63	0.49, 0.81	<0.001
**Obesity**	1,197	0.60	0.47, 0.77	<0.001
**History of depression**	1,205	0.61	0.45, 0.83	0.002
**Chronic kidney disease**	1,204	0.34	0.16, 0.74	0.007
**SF12 quality of life physical health score**	1,205	1.05	1.03, 1.07	<0.001
**SF12 quality of life mental health score**	1,205	1.03	0.99, 1.07	0.125

[1] **OR = Odds Ratio, CI = Confidence Interval**

Note: Exercise criteria defined as a GPAQ score of greater or equal to 600 at 12 months post- discharge. This is equivalent to 75 minutes of vigorous activity or 150 minutes of moderate activity or a combination of both.

Abbreviations used: OR = odds ratio, CI = confidence interval, BMI = body mass index, BP = blood pressure, LDL = low-density lipoprotein, HDL = high-density lipoprotein, SF12 = 12-item Short Form Survey

Supplementary Table 6. Univariate logistic regression for possible association of covariates with smoking status outcome.

Covariates	N	OR[1]	95% CI[1]	p-value
**Ethnicity (Caucasian)**	1,248	0.90	0.63, 1.32	0.579
**>=10 standard drinks per week**	1,247	1.43	1.00, 2.01	0.052
**Employed full-time/part-time**	1,247	1.30	0.95, 1.80	0.096
**Education Level**	1,245			<0.001
**Year 10 or under**		—	—	
**Year 12**		0.57	0.36, 0.87	
**University/Diploma or above**		0.46	0.33, 0.64	
**Married/Defacto**	1,248	0.50	0.37, 0.69	<0.001
**Income greater than 1,000 AUD/week**	1,247	0.65	0.48, 0.89	0.007
**Physical disability**	1,248	0.92	0.40, 1.88	0.836
**Prior cardiovascular events**	1,248	0.89	0.63, 1.23	0.474
**Obesity**	1,240	1.01	0.74, 1.38	0.941
**History of depression**	1,248	1.83	1.28, 2.60	0.001
**SF12 quality of life physical health score**	1,248	1.01	0.99, 1.04	0.283
**SF12 quality of life mental health score**	1,248	1.01	0.97, 1.07	0.640
**Exercising regularly**	1,241	1.11	0.81, 1.54	0.507

[1] **OR = Odds Ratio, CI = Confidence Interval**

Abbreviations used: OR = odds ratio, CI = confidence interval, BMI = body mass index, BP = blood pressure, LDL = low-density lipoprotein, HDL = high-density lipoprotein, SF12 = 12-item Short Form Survey
